# Should Prior FIT Results Be Incorporated as an Additional Variable to Estimate Risk of Colorectal Neoplasia? A Prospective Study of 5,813 Screening Colonoscopies

**DOI:** 10.1371/journal.pone.0114332

**Published:** 2014-12-05

**Authors:** Martin C. S. Wong, Jessica Y. L. Ching, Victor C. W. Chan, Jeffrey P. Shum, Thomas Y. T. Lam, Arthur K. C. Luk, Joseph J. Y. Sung

**Affiliations:** 1 Institute of Digestive Disease, Faculty of Medicine, Chinese University of Hong Kong, Hong Kong, China; 2 School of Public Health and Primary Care, Faculty of Medicine, Chinese University of Hong Kong, Hong Kong, China; The University of Texas MD Anderson Cancer Center, United States of America

## Abstract

**Background:**

Recent studies showed that previous negative results from faecal immunochemical tests (FITs) for colorectal cancer (CRC) screening was associated with lower risk of advanced neoplasia (AN). We evaluated whether prior FIT results should be included to estimate the risk of AN in 2008–2012.

**Methods:**

A community-based screening practice recruited 5,813 asymptomatic residents aged 50 to 70 years in Hong Kong for CRC screening. We included study participants who had (1). positive FIT with subsequent colonoscopy workup (FIT+ group; n = 356); (2). negative FIT in three consecutive years and received a colonoscopy (FIT- group; n = 857); (3). received colonoscopy without FIT (colonoscopy group; n = 473); and (4). received both colonoscopy and FIT at the same time (combined group; n = 4,127). One binary logistic regression model evaluated whether prior FIT results were associated with colonoscopy findings of AN.

**Results:**

The proportion of participants having AN/CRC was 18.0% (FIT+), 5.5% (FIT-), 8.0% (colonoscopy group), and 4.3% (combined group), respectively. When compared with the colonoscopy group, those in the FIT- group were not significantly more or less likely to have AN/CRC (AOR  = 0.77, 95% C.I. = 0.51 to 1.18, p  = 0.230). Having one (AOR = 0.73, 95% C.I. 0.48–1.12, p = 0.151) or three consecutive negative FIT result (AOR = 0.98, 95% C.I. 0.60–1.62, p = 0.944) were not associated with lower risks of AN/CRC. Subjects in the FIT+ group was 3.32-fold (95% C.I. 2.07 to 5.32, p<0.001) more likely to have AN/CRC.

**Conclusions:**

These findings indicated that subjects with negative FIT findings could be risk stratified similarly as those who had not previously received FIT.

## Introduction

Colorectal cancer (CRC) has been a leading cause of morbidity and mortality worldwide. It is the third most common cancer in men and second in women, attributable to more than 10% of all malignancies and 8% of cancer deaths globally [1]. Apart from its high incidence in many western countries like the US, Canada and the European nations, the past decade has also witnessed a two- to three-fold rise in Asia Pacific countries including China, Japan, Korea, Singapore and Hong Kong [2]. It has been estimated that the direct medical costs for care of CRC approximate to more than US$44,000 at stage IV, contributing to a substantial global health burden [3].

CRC screening by Faecal occult blood tests (FOBT) and colonoscopy could effectively reduce CRC mortality by 33% and 56%, respectively [4], [5]; and these two tests were the most commonly used screening modalities. International guidelines and expert consensus statements recommend CRC screening for average risk subjects aged over 50 years, including the Asia Pacific countries [6], [7]. Currently, both FOBT and colonoscopy are roughly equally accepted as first-line screening tests [8]. In underprivileged countries or regions where colonoscopic capacity is a concern [9]–[11], FOBT may be more viable as an option – hence colonoscopy screening should be reserved for higher risk subjects to optimize resource utilization, especially for population-based screening. Primary care physicians will need to risk stratify their patients to determine the most appropriate screening tool.

There are a number of clinical risk scores devised to predict the risk for colorectal neoplasia [12]–[17]. The Asia Pacific Colorectal Screening (APCS) scoring system [12] was based on age, gender, family history and smoking habits to assign risk for individuals. It enables risk stratification using elementary clinical information, and successfully predicts the risk of colorectal advanced neoplasia (AN) in asymptomatic subjects. The scoring system recommended colonoscopy screening for high-risk subjects, and average-risk subjects for faecal tests [12]. Four other scoring systems recruited in Germany [13], the United States [14]–[16], and Spain [17] similarly based on a subject's risk to inform the most preferred screening tool.

However, there is a recent study which investigated whether giving Faecal Immunochemical Tests (FITs) between surveillance colonoscopies may offer additional benefit for earlier detection of AN [18]. Lane and colleagues (2010) found that patients who had repeated negative results from FIT had around 2-fold reduced risk for cancer and AN [18], and facilitated earlier diagnoses of CRC and AN by 25 months and 24 months, respectively. The FIT result was also predictive for the neoplastic stage of AN, and the probability of the most advanced neoplasia was lowest with a negative result from FIT (odds ratio = 0.68). Therefore, it is reasonable to speculate whether priori FIT result could act as an additional variable when a subject is risk stratified for CRC screening.

Thus far, this knowledge gap has not been addressed. Knowing this is important because most population-based CRC screening programme relies on FIT as the initial test. As one ages the predicted risks increase with time. Taking the APCS scoring system as an example, there is a 2-point increment when one reached 50 year-old and an additional 1-point increment when reached 70 [12]. For screening participants who are initially classified as average risk (i.e. FIT recommended), they will, at a future time point, be stratified as high risk (i.e. colonoscopy indicated).

The objective of this study is to evaluate whether priori FIT results should be considered as an additional piece of information to risk stratify subjects for CRC screening. We tested the *a priori* hypothesis that priori FIT results should be used as a predictive variable on top of the APCS scoring system, based on findings from Lane and colleagues [18].

## Materials and Methods

### Settings

The setting of this study has been described elsewhere [8], [19]–[24]. A primary care screening centre was established in Hong Kong in 2008. Free CRC screening was provided for all eligible Hong Kong residents. Subjects were recruited via media invitations or referrals by physicians in clinics affiliated with the screening centre. This community centre provides education and CRC screening to the entire population of Hong Kong, and is accessible to all citizens. We collected data based on screening recruitment between 2008 and 2012, and the data were analyzed in 2013. All subjects gave written informed consent prior to study enrolment. This study was approved by the Clinical Research Ethics Committee of the Chinese University of Hong Kong, and has been performed in accordance with the ethical standards laid down in the 1964 Declaration of Helsinki and its later amendments.

### Recruitment of Participants

Eligible subjects included adults aged between 50–70 years who were asymptomatic of CRC, including per rectal bleeding, unexplained anemia, change in bowel habit in the past 4 weeks, or weight loss of greater than 5 kg in the past 6 months. The exclusion criteria included: (1). past history of colorectal diseases which may increase the risks of CRC, like colorectal neoplasm and inflammatory bowel disease; (2). History of examination of colon in the past 5 years, including colonoscopy, barium enema, and imaging tests; (3). Severe premorbid illnesses that increase the risk of colonoscopy, like cardiopulmonary insufficiency, bleeding disorders, cirrhosis, cardiac surgery and the use of anticoagulants or double antiplatelet agents; (4). History of colorectal surgery; and (5). The presence of contraindications for colonoscopy.

### Study logistics

The subjects identified as eligible were explained about the nature and purpose of the study, and informed consents were obtained. Their demographic and clinical details were collected, including age, gender, smoking and drinking habits, history of CRC among their first-degree relatives, past medical history, and long-term medication use. Body weight was measured with the participant wearing light clothing using reliable weight scales, which were regularly calibrated over time. The body height was assessed by a stadiometer with the subject not wearing shoes. All participants were then offered an educational session using a standard video followed by health talks on CRC and its screening by trained educators. All educators were trained by a team of gastroenterologists, family physicians and public health professionals before the programme. The participants received either a yearly FIT or one direct colonoscopy. This study included participants who received colonoscopy. They were in one of the following groups: (1). “FIT+ve group” (n = 356): subjects who chose yearly FIT, and received a colonoscopy because at least one FIT was positive (at any year); (2). “FIT-ve group” (n = 857): subjects who chose FIT, had negative FIT results in all subsequent three consecutive years; and invited for surveillance colonoscopy at year three; (3). Colonoscopy group (n = 473): subjects who chose colonoscopy in the first year; (4). Combined group (n = 4,127): Subjects who received both FIT and colonoscopy in the first year, irrespective of the FIT results.

### Faecal immunochemical tests and colonoscopic procedures

Participants who underwent a FIT received information on procedures for completing the FIT at home, according to the manufacturer's instructions (Hemosure; W.H.P.M., Inc, El Monte, CA, USA). Each participant was requested to poke the spiral applicator into six different sites of their stool specimen – which was just enough to cover the tip of the applicator. The applicator was then screwed back into the sample collection tube and secured tightly. They were returned by the screening participants to the centre for further analysis. All FITs were tested within 48 hours of receipt by trained professionals. For each test, three drops of test solutions were squeezed from the collection tube into the sample well. The test results were read 5 minutes afterwards. A reminder sheet listing the dates of yearly return was issued for the participants. They were requested to return the specimens and collection tubes to the centre within 6 days of the expected dates of their return, on a yearly basis. Up to three separate telephone reminders were sent to those participants who had not returned the collection tubes on the expected dates. Those who were non-adherent to the FIT were offered FIT again in subsequent years.

Participants who received colonoscopy were explained about the procedure and a telephone appointment reminder one week before the scheduled endoscopy appointment. A standardized bowel preparation regime using Polyethylene Glycol (Klean-Prep^R^, Helsinn Birex Pharmaceuticals Ltd, Ireland) was given to each participant before they left the centre. Prior to the colonoscopy, all subjects received a standard sedation regime consisting of Midazolam 2.5mg (Groupe Panpharma, France). Pethidine 25 mg (Martindale Pharmaceuticals, United Kingdom) was administered intravenously. Further doses of Midazolam and Pethidine were given according the subject's level of discomfort. Air insufflation was used for all the colonoscopies in this study. A withdrawal time of at least 6 minutes was practiced for all subjects, which is in accordance with the current quality indicators for colonoscopy [25]. Conventional white light colonoscopy was performed by experienced colonoscopists under conscious sedation. The colonoscopic findings, including caecal intubation time and the adequacy of bowel preparation, were documented. All lesions were removed and biopsied as deemed appropriate by the endoscopists. The biopsied specimens were examined by gross and microscopic evaluation in a certified laboratory by experienced histopathologists. Advanced neoplasia was defined as CRC, or any colorectal adenoma which had a size of ≥ 10 mm in diameter, high-grade dysplasia, villous or tubulovillous histologic characteristics, or any combinations thereof [26].

### Statistical Analyses

All data were entered into a predesigned database with logistic checking using Microsoft Access, and analyzed using SPSS software, version 18.0 (Chicago, Illinois). Participant characteristics were compared among the four groups aforementioned. The outcome variable is the colonoscopy finding of AN. The variable tested for association with AN was the FIT result (i.e. positive for one year; negative for one year; negative for three years). Variables recognized as risk factors for CRC were tested for univariate analysis, and those variables with initial p<0.10 were entered into a final binary logistic regression model. These included age, gender, BMI, smoking, alcohol drinking, diabetes mellitus, family history of CRC, self-reported hypertension, heart diseases, and use of NSAIDs or aspirin. Secondly, the same regression model was re-constructed with the “colonoscopy group” as the reference, whilst all participants having positive FIT or negative FIT (irrespective of the number of years tested) were evaluated for association. Finally, the same regression model was analyzed comparing negative FIT for one vs. three consecutive years. All p values <0.05 were considered statistically significant.

## Results

### Participant characteristics

From 5,813 screening participants, the average age was 57.7 years (SD 4.9) with a female proportion of 53.1% (**Table 1**). The majority were non-smokers (92.1%) and non-drinkers (90.3%). 14% had a first-degree relative with past history of CRC. The proportion of subjects having hypertension, diabetes and cardiovascular diseases was 23.0%, 7.5% and 1.7%, respectively. Among them, 4.6% and 2.4% reported long-term use of NSAIDs and aspirin, respectively (**Table 1**). A total of 22 cancers were detected, and 5.3% had AN.

**Table 1:** Participant characteristics (N = 5,813).

	All subjects (N = 5,813)	FIT+ve group (n = 356)	FIT-ve group (n = 857)	Colonoscopy group (n = 473)	Combined group (n = 4,127)	P values
Age (years), mean±SD	57.7 (4.9)	59.3 (5.3)	57.7 (5.0)	58.1 (5.3)	57.5 (4.8)	<0.001
BMI (kg/m^2^), mean±SD	23.5 (3.2)	23.9 (3.3)	23.7 (3.3)	23.7 (3.3)	23.5 (3.1)	<0.001
Gender, n (%)						<0.001
Male	2,727 (46.9)	166 (46.6)	358 (41.8)	326 (68.9)	1,877 (45.5)	
Female	3,086 (53.1)	190 (53.4)	499 (58.2)	147 (31.1)	2,250 (54.5)	
Current Smoking, n (%)						<0.001
Non-smoker	5,353 (92.1)	335 (94.1)	823 (96.0)	254 (53.7)	3,941 (95.5)	
Ex-smoker/smoker	460 (7.9)	21 (5.9)	34 (4.0)	219 (46.3)	186 (4.5)	
Alcohol drinking, n (%)						<0.001
Non-drinker	5,252 (90.3)	313 (87.9)	791 (92.3)	376 (79.5)	3,772 (91.4)	
Drinker/ex-drinker	561 (9.7)	43 (12.1)	66 (7.7)	97 (20.5)	355 (8.6)	
Diabetes mellitus, n (%)	434 (7.5)	26 (7.3)	71 (8.3)	41 (8.7)	296 (7.2)	<0.001
Family history present for a first-degree relative, n (%)	815 (14.0)	41 (11.5)	101 (11.8)	161 (34.0)	512 (12.4)	<0.001
Hypertension, n (%)	1,336 (23.0)	97 (27.2)	205 (23.9)	127 (26.8)	907 (22.0)	<0.001
IHD/Heart Disease, n (%)	98 (1.7)	5 (1.4)	10 (1.2)	15 (3.2)	68 (1.6)	<0.001
Use of NSAIDs, n (%)	269 (4.6)	17 (4.8)	39 (4.6)	38 (8.0)	175 (4.2)	<0.001
Use of Aspirin, n (%)	139 (2.4)	15 (4.2)	16 (1.9)	3 (0.6)	105 (2.5)	<0.001
**Colonoscopic findings**						
Colorectal Cancer, n (%)	22 (0.4)	8 (2.2)	0 (0.0)	2 (0.4)	12 (0.3)	<0.001
Advanced Neoplasia, n (%)	306 (5.3)	56 (15.7)	47 (5.5)	36 (7.6)	167 (4.0)	<0.001

*Advanced Neoplasia is defined as any colorectal adenoma which has a size of ≥ 10 mm in diameter, high grade dysplasia, villous or tubulovillous histologic characteristics, or any combination thereof. BMI: Body Mass Index; FIT: Faecal Immunochemical Tests; IHD: Ischemic Heart Disease; NSAIDs: Non-steroidal Anti-Inflammatory Disease.

The colonoscopy group had significantly greater proportion of males (68.9% vs. 46.9% overall); smokers or ex-smokers (46.3% vs. 7.9%); drinkers or ex-drinkers (20.5% vs. 9.7%); diabetes (8.7% vs. 7.5%); first-degree relatives with CRC (34.0% vs. 14.0%) and cardiovascular diseases (3.2% vs. 1.7%), when compared with all subjects. This group was therefore at the highest risks for AN (8.0% vs. 5.7%). The FIT-ve group had lower proportions of smokers or ex-smokers (4.0% vs. 7.9%) and cardiovascular diseases (1.2% vs. 1.7%). They were at the lowest risks for CRC (0.0% vs. 0.4% overall) (**Table 1**).

### Association between risk factors and colorectal advanced neoplasia

From univariate analysis, it was found that advanced age (adjusted odds ratio [AOR] =  1.08); higher BMI (AOR = 1.08); male gender (AOR = 1.91); current smoking (AOR = 1.83); alcohol drinking (AOR = 1.80); family history of CRC (AOR = 1.50) and self-reported hypertension (AOR = 1.78) were significantly associated with AN/CRC (**Table 2**). When compared with the colonoscopy group and combined group, those with positive FIT for one year was 4.43 times (95% C.I. 3.27–5.99, p<0.001) more likely to have AN. Having negative FIT for three years was not associated with the outcome (AOR = 1.17, 95% C.I. 0.85–1.62, p = 0.337).

**Table 2:** The association between prior FIT findings and colonoscopic findings of advanced neoplasia/CRC.

	Crude odds ratio	p	Adjusted odds ratio	p
Age (years), mean±SD	1.08 (1.06–1.10)	<0.001	1.06 (1.04–1.09)	<0.001
BMI (kg/m^2^), mean±SD	1.08 (1.04–1.11)	<0.001	1.05 (1.01–1.09)	0.009
Gender, male, n (%)	1.91 (1.52–2.40)	<0.001	1.61 (1.25–2.07)	<0.001
Current Smoking, n (%)	1.83 (1.31–2.57)	<0.001	1.45 (1.00–2.09)	0.048
Alcohol consumption, n (%)	1.80 (1.31–2.45)	<0.001	1.31 (0.93–1.83)	0.122
Diabetes mellitus, n (%)	1.32 (0.90–1.93)	0.160	–	–
Family history present for a first-degree relative, n (%)	1.50 (1.13–1.99)	0.006	1.54 (1.15–2.08)	0.004
Hypertension, n (%)	1.78 (1.40–2.26)	<0.001	1.39 (1.07–1.80)	0.013
IHD/Heart Disease, n (%)	1.50 (0.72–3.12)	0.279	–	–
Use of NSAIDs, n (%)	0.64 (0.33–1.21)	0.165	–	–
Use of Aspirin, n (%)	1.60 (0.88–2.93)	0.125	–	–
**CRC screening groups**				
Colonoscopy group and combined group	1.00 (Referent)		1.00 (Referent)	
FIT+ in the first year	4.43 (3.27–5.99)	<0.001	4.01 (2.92–5.51)	<0.001
FIT-ve for three years	1.17 (0.85–1.62)	0.337	1.23 (0.88–1.73)	0.225

*The n number and % represent the number and proportion (across rows) of subjects found to have advanced neoplasia or colorectal cancer on colonoscopy. Advanced Neoplasia is defined as any colorectal adenoma which has a size of ≥ 10 mm in diameter, high grade dysplasia, villous or tubulovillous histologic characteristics, or any combination thereof. BMI: Body Mass Index; CRC: colorectal cancer; FIT: Faecal Immunochemical Tests; IHD: Ischemic Heart Disease; NSAIDs: Non-steroidal Anti-Inflammatory Disease.

In multivariate regression analysis, all the risk factors except alcohol drinking (p = 0.122) remained significant (p<0.05). Positive FIT result for one year was associated with higher odds (AOR = 4.01, 95% C.I. 2.92–5.51, p<0.001) of AN; whereas there was no association between negative FIT for three years and the colonoscopic outcomes (AOR = 1.23, 95% C.I. 0.88–1.73, p = 0.225).

### Subgroup analyses

When compared with the colonoscopy group, those with positive FIT results were associated with higher odds of having AN in both univariate (18.3% vs. 8.0%; OR = 2.56, 95% C.I. 1.69–3.87, p<0.001) and multivariate analyses (OR = 3.32, 95% C.I. 2.07–5.32, p<0.001) (**Table 3**). Those having negative FIT results for either one or three years were significantly less likely to have AN (4.3% vs. 8.0%, AOR = 0.52, 95% C.I. 0.36–0.75, p<0.001) in univariate analysis. However, when the significant risk factors were controlled in multivariate regression analysis, both negative FIT result for one year (AOR = 0.73, 95% C.I. 0.48–1.12, p = 0.151) and for three years (AOR = 0.98, 95% C.I. 0.60–1.62, p = 0.944) were not significantly associated with fewer colonoscopic outcomes.

**Table 3:** The association between CRC screening groups and the colonoscopic findings of advanced neoplasia/CRC.

CRC screening groups – analysis (1)						
	n	%	Crude odds ratio	p	Adjusted odds ratio	p
Colonoscopy group (n = 473)	38	8.0	1.00 (Referent)		1.00 (Referent)	
FIT+ve in the first year plus FIT+ve in the combined group (n = 416)	76	18.3	2.56 (1.69–3.87)	<0.001	**3.32 (2.07–5.32)**	**<0.001**
FIT-ve for three years plus FIT-ve in the combined group (n = 4,924)	214	4.3	0.52 (0.36–0.75)	<0.001	**0.77 (0.51–1.18)**	**0.230**
**CRC screening groups – analysis (2)**						
Colonoscopy group (n = 473)	38	8.0	1.00 (Referent)		1.00 (Referent)	
FIT+ve in the first year plus FIT+ve in the combined group (n = 416)	76	18.3	2.56 (1.69–3.87)	<0.001	**3.32 (2.07–5.32)**	**<0.001**
FIT-ve in the combined group (n = 4,067)	167	4.1	0.49 (0.34–0.71)	<0.001	**0.73 (0.48–1.12)**	**0.151**
FIT-ve for three years (n = 857)	47	5.5	0.66 (0.42–1.04)	0.070	**0.98 (0.60–1.62)**	**0.944**

The adjusted model controlled for age, gender, BMI, smoking, alcohol intake, family history of CRC and self-reported hypertension. n (%) represents the number and proportion of patients having advanced neoplasia or CRC for each row.

## Discussion

### Statement of principal findings

This study compared the risks for AN among those having no prior FIT results, positive FIT findings and negative FIT findings. It was found that those having negative FIT results, no matter for a single year or three consecutive years, were less likely to suffer from AN. However, these significantly lower associations with AN disappeared after the recognized risk factors for CRC were taken into account. These findings do not support the use of prior FIT results to risk stratify subjects for CRC screening when validated risk scoring system is already in place.

### Relationship with literature and Explanations of study findings

From a literature search, there has been no study which evaluated the risk of AN in the presence of FIT results, except the Dutch FOBT-based screening pilot [27]. It compared the incidence of AN in the second year of the programme between those tested negative vs. positive in the first year. From 4,200 participants who joined the first two rounds of the screening programme, a significant reduction in the positive predictive value (PPV) for advanced neoplasia from 55% to 44% (p = 0.017) was observed, as was CRC (from 8% to 4%, p = 0.024). This is compatible with what we have reported here. These could not, however, be directly compared with the findings of this study, as the Dutch cohort who was tested negative for FIT did not receive colonoscopy, nor were they followed for a period of three years. Our study is also unique as we further explored the predictive values of prior FIT results in the context of other risk factors.

The proportions of subjects having AN or CRC were the lowest among subjects in the combined group. This group had the lowest average age (57.5 vs. 57.7 years) and BMI (23.5 vs. 23.7–23.9 kg/m^2^) when compared with other subjects. There were fewer male subjects; smokers; alcohol drinkers – and the proportion of screening participants suffering from self-reported diabetes or hypertension was also lower. These may explain the lower prevalence of AN/CRC.

We found that priori negative FIT for one or three consecutive years was not associated with significantly lower risks for AN when other risk factors were controlled in the multivariate regression model. There are several explanations of this observation. Firstly, the most important covariates including age, gender, family history of CRC, smoking, alcohol intake, and self-reported hypertension were all well-established risk factors. There left little room for additional variables to account for the variability of the outcome, as covariates in a regression model tend to compete with each other to predict the outcome variable. Secondly, the sensitivity of FIT in detecting advanced neoplasia and cancer in our previous analysis was only 35.1% (95% C.I. 20.7%–52.6%) and 25.0% (95% C.I. 1.3%–78.1%) [22]. Therefore, even if we take into account the compounding effect of negative results accumulated over three years, the 3-year pooled sensitivity might still be low. A recent systematic review [28] showed that the pooled sensitivity of FITs for CRC was only 79% (95% C.I. 69%–86%), and there were no significant differences in the performance characteristics among various commercial FIT brands in general.

### Limitations

This study included a relatively large number of asymptomatic screening participants. There are yet limitations which should be addressed here. Firstly, the analysis is based on self-selected cohorts, who might be more health-conscious as compared to the population. Its representativeness to the general public could be limited, but simple random sampling might be problematic as the proportion of refusal will be high, based on results from a population-based survey in Hong Kong [29]. In addition, we have not adopted a randomized design when assigning these participants into the different groups. One might argue that members in the “colonoscopy group” had baseline characteristics which render them to carry higher risk for AN. Nevertheless, it is practically difficult to allocate a single option to each screening participant, as it will lower their adherence with screening over time if no choice was offered [30]. Besides, the inherently higher risks of subjects in the colonoscopy group should theoretically bias the findings towards a significantly lower risk of AN in the FIT negative group after confounder adjustment – yet this was not the case. Also, we regarded colonoscopy as the gold standard for comparison, and it is well recognized that missed lesions do occur. The overall miss rates for neoplastic lesions ranged from 8% to 24%, especially high for those polyps small than 10 mm and those located at the splenic flexure and the caecum [31]–[33]. Finally, this study used a qualitative FIT, which expresses cutoff hemoglobin concentrations using a range of units - and there exists no requirement for commonality in methodologic principles and procedures of standardization [34].

### Conclusions

In summary, patients attending clinical practices with previous negative test results, be it for a single year or three year, were found to have much lower risks for advanced lesions or cancer. Existing scoring systems for prediction of colorectal neoplasia do not incorporate priori faecal test results as an independent variable. Therefore, given the findings of the present study, physicians may not need to incorporate faecal test results for risk stratification if their practices are already using validated tools to classify their patients into different risk categories, such as the APCS scoring system. Nevertheless, we recommend physicians to base on risk estimates to communicate with the screening participants on their individual risks for AN, so that a fully informed choice on the screening modality used could be made.
